# Enterovirus genotypes causing hand foot and mouth disease in Shanghai, China: a molecular epidemiological analysis

**DOI:** 10.1186/1471-2334-13-489

**Published:** 2013-10-22

**Authors:** Menghua Xu, Liyun Su, Lingfeng Cao, Huaqing Zhong, Niuniu Dong, Jin Xu

**Affiliations:** 1 Laboratory Medicine Center, Pediatric Institute, Children’s Hospital of Fudan University, Shanghai 201102, China

**Keywords:** Hand foot and mouth disease (HFMD), Human enterovirus (HEV), Genotype

## Abstract

**Background:**

A rapid expansion of hand, foot, and mouth disease (HFMD) outbreaks has occurred and caused deaths in China in recent years, but little is known about the other etiologic agents except enterovirus 71 (EV71) and coxsackievirus A 16 (CA16). The objective of this study is to determine the genotype compositions of enterovirus causing HFMD in Shanghai and identify any associations between enterovirus types and clinical manifestations.

**Methods:**

Stool specimens were collected from patients hospitalized for treatment of HFMD, from May 2010 to April 2011. Enterovirus was detected by reverse transcription PCR and directly genotyped by sequencing the PCR products. Phylogenetic analysis was based on the VP1 partial gene.

**Results:**

Of 290 specimens, 277 (95.5%) tested positive for enterovirus. The major genotypes were EV71 (63.8%), CA10 (9.0%), CA6 (8.3%), CA16 (6.9%), CA12 (2.4%), and CA4 (1.4%). The EV71 strains belonged to the C4a subtype and CA16 belonged to the B subtype. CA6 was closely related to strains detected in Japan, Taiwan and China, and CA10, CA12 and CA4 were phylogenetically similar to other strains circulating in China. Mean hospital stays and the prevalence of complications in patients with EV71 infection were higher than those in patients in CA6, CA10 or CA16 infection (*P* < 0.05 for all comparisons). Children with CA12 infection were the youngest, and most likely have the highest risk of complications when compared to the other non-EV71 infection groups.

**Conclusions:**

This study demonstrated a diversified pathogen compositions attributing to HFMD and clinical symptoms differing in enterovirus genotypes. It deserves our attention as early identification of enterovirus genotypes is important for diagnosis and treatment of HFMD patients.

## Background

Hand, foot, and mouth disease (HFMD), first reported in New Zealand in 1957, is a global and common infectious disease in young children, particularly in those less than 5 years old. Vesicular exanthema develops on the hands, feet, mouth and buttocks. The disease usually resolves spontaneously, but severe complications, including death, can occur [1-4]. Large-scale outbreaks have occurred in China and caused 126 and 353 deaths in the year of 2008 and 2009, respectively (http://www.chinacdc.cn/tjsj/fdcrbbg/201002/t20100224_25293.htm) [1-7].

Hand, foot, and mouth disease is caused by human enterovirus, a genus of the *Picornavirus* family, and is characterized by a single 7.4kb of positive-strand genomic RNA. The genomes contain only a single long open reading frame that encodes four structural proteins (VP1 through VP4) and seven non-structural proteins (2A, 2B, 2C, 3AB, 3C and RNA-dependent RNA polymerase 3D), which are responsible for viral replication and protein processing. The VP1 is the most external and immunodominant *picornavirus* capsid protein and contains the major neutralization epitopes that has been used for virus serotype identification and evolutionary studies [8-10].

Enterovirus genotypes are classified into four species, A, B, C and D, on the basis of genome organization, sequence similarity and biological properties [11]. Enterovirus 71 (EV71) and coxsackievirus A16 (CA16) belonging to species A are the most common causes of HFMD worldwide [1]. Severe EV71-associated clinical syndromes, such as acute flaccid paralysis, brainstem encephalitis, rapid fatal pulmonary edema, and hemorrhage, have been observed in many outbreaks and is considered an important cause of severe HFMD [2,3]. CA16 associated HFMD has a milder outcome and a much lower incidence of severe complications [12].

Though HFMD has become a public issue in China, little is known about the other etiologic agents except EV71 and CA16. Sporadic reports demonstrated that various enterovirus genotypes, such as CA2, CA4, CA5, CA6, CA8, CA10 and CA12 from species A, CB2 and CB4 from species B and some echoviruses can cause HFMD [13-17]. However, studies on the relationship of these genotypes with clinical symptoms are lacking.

In this study, we aimed to investigate the genotypes of enterovirus contributing to HFMD and to identify any associations between enterovirus types and clinical manifestations in Shanghai, China.

## Methods

### Sample collection

From 1 May 2010 to 30 April 2011, stool specimens were collected from patients hospitalized for HFMD at children’s Hospital of Fudan University, Shanghai, China. All the patients were diagnosed by the Ministry of Health diagnostic criteria (http://www.nhfpc.gov.cn/yzygj/s3593g/201306/6d935c0f43cd4a1fb46f8f71acf8e245.shtml). The complications involve diseases in neurologic, respiratory or circulatory system that caused by HFMD, such as aseptic meningitis, encephalitis, acute flaccid paralysis, pulmonary oedema or cardiorespiratory failure, which were defined in “A Guide to Clinical Management and Public Health Response for Hand, Foot and Mouth Disease (HFMD)” (http://www.wpro.who.int/publications/docs/GuidancefortheclinicalmanagementofHFMD.pdf).

Patients’ demographic data, clinical symptoms, and major complications were collected retrospectively from medical history. This study was approved by the Ethics Committee in the Children’s Hospital of Fudan University. Because the specimens were collected in the normal course of patient care, no informed consent was required according to the Ethics Committee.

### Human enterovirus testing

From supernatants of 10% (V/V) stool specimens, RNA was extracted by Trizol (Invitrogen, CA, USA) according to the manufacturer’s instructions. The RNA was dissolved in 20 μL DEPC (Diethypyrocarbonate) water. cDNA was synthesized using 4 μL of extracted RNA, 100 μmol of random primers, and 2.5 U of reverse transcriptase (PrimeScript TM RT kit, Takara, Dalian, China). The reverse transcriptase reaction was carried out at 37°C for 30 min and 85°C for 5 s.

Human enterovirus was preliminarily detected with highly conserved 5′UTR primers. The first PCR step was performed in a reaction volume of 25 μL, including 2 μL cDNA, 0.5 μmol each of outer primers (EV1F: 5′-CGGCCCCTGAATGCGGC-3′, EV1R: 5′-CACCGGATGGCCAATCCA-3′), 50 μmol dNTP, and 0.75U of ExTaq DNA polymerase (Takara, Dalian, China). The PCR protocol was to use an initial temperature of 94°C for 1 min, followed by 35 cycles of 94°C for 30 s, 55°C for 30s, 72°C for 30 s, and 72°C for 7 min. Then 1 μL of DNA from the first round of PCR was used as the template in the second round of PCR with the inner primers (EV2F: 5′-CCCCTGAATGCGGCTAAT-3′, EV2R: 5′-ATTGTCACCATAAGCAGCCA-3′) under the same reaction system and cycling conditions. The PCR products were subjected to electrophoresis in a 2% agarose gel to identify positive samples with a predicted size of 146 bp.

### Enterovirus genotyping

For enterovirus genotyping, samples tested positive by the 5′UTR were amplified by nested PCR using primers from the VP1 junction region of enterovirus as described [18]. For the first round of PCR, 2 μL of cDNA was used in a volume of 25 μL containing 0.5 μmol each of outer primers, 50 μmol of dNTP, and 0.75U of ExTaq DNA polymerase in the following cycling conditions: 35 cycles of 94°C for 30 s, 54°C for 45 s, 72°C for 30 s, and a final incubation of 72°C for 7 min. A volume of 2 μL of the product was used for the second round of PCR with 0.5 μmol each of inner primers, 50 μmol of dNTP, and 0.75U of ExTaq DNA polymerase in a volume of 25 μL undergoing the same PCR conditions as described above. The final product of the different groups of enterovirus was visualized by agarose gel electrophoresis. DNA fragments were purified and the nucleotide sequence of each PCR product was bi-directional sequenced on a 3730 sequencer (Pekin-Elmer Applied Biosystems, Foster City, CA). The PCR products used for the sequencing were about 683 bp for the group A, 619 bp for the group B and 497 bp for the 5′UTR.

### Phylogenetic analysis and sequence submission

Sequence analysis of the PCR product of each strain was analyzed with Seqscanner software (Applied Biosystems, USA), and genetic identity was determined by comparing the sequence with standard strains in Genbank (US National Center for Biotechnology Information, NCBI). A multiple-sequence alignment was constructed using ClustalW and phylogenetic trees were performed applying the neighbor-joining method with the 1000-bootstrap re-sampling implemented in the Molecular Evolutionary Genetics Analysis, version 5.0 program. All the VP1 gene sequences were submitted to GenBank sequence database (NCBI, Betheseda, MD, USA) with given accession numbers KC834832-KC834854 for CA6, KC834855-KC834877 for CA10, KC834878-KC834892 for CA16 and KC834893-KC835055 for EV71. The phylogenetic trees have been deposited in TreeBASE (# 14766).

### Statistical methods

The means of the continuous variables and the proportions of the categorical variables were compared using Student’s *t* test, *x*^2^ tests, and Fisher’s exact test, as appropriate. All data met the assumptions of the tests used to analyze them. Alpha was set at 0.05. Data analysis was performed using SPSS version 17.0 for windows.

## Results

### Enterovirus genotypes

Of 290 specimen, 277 (95.5%) tested positive for enterovirus. Except for 8 specimens that could not be typed, nine different genotypes were identified with different detection rates: EV71 (63.8%, 185/290), CA10 (9.0%, 26/290), CA6 (8.3%, 24/290), CA16 (6.9%, 20/290), CA12 (2.4%, 7/290), CA4 (1.4%, 4/290), CA14 (0.3%, 1/290), Echo6 (0.3%, 1/290), and HEV-C (0.3%, 1/290). No mixed infections were found.

### Phylogenetic analysis of enterovirus genotypes

**Figure 1:**
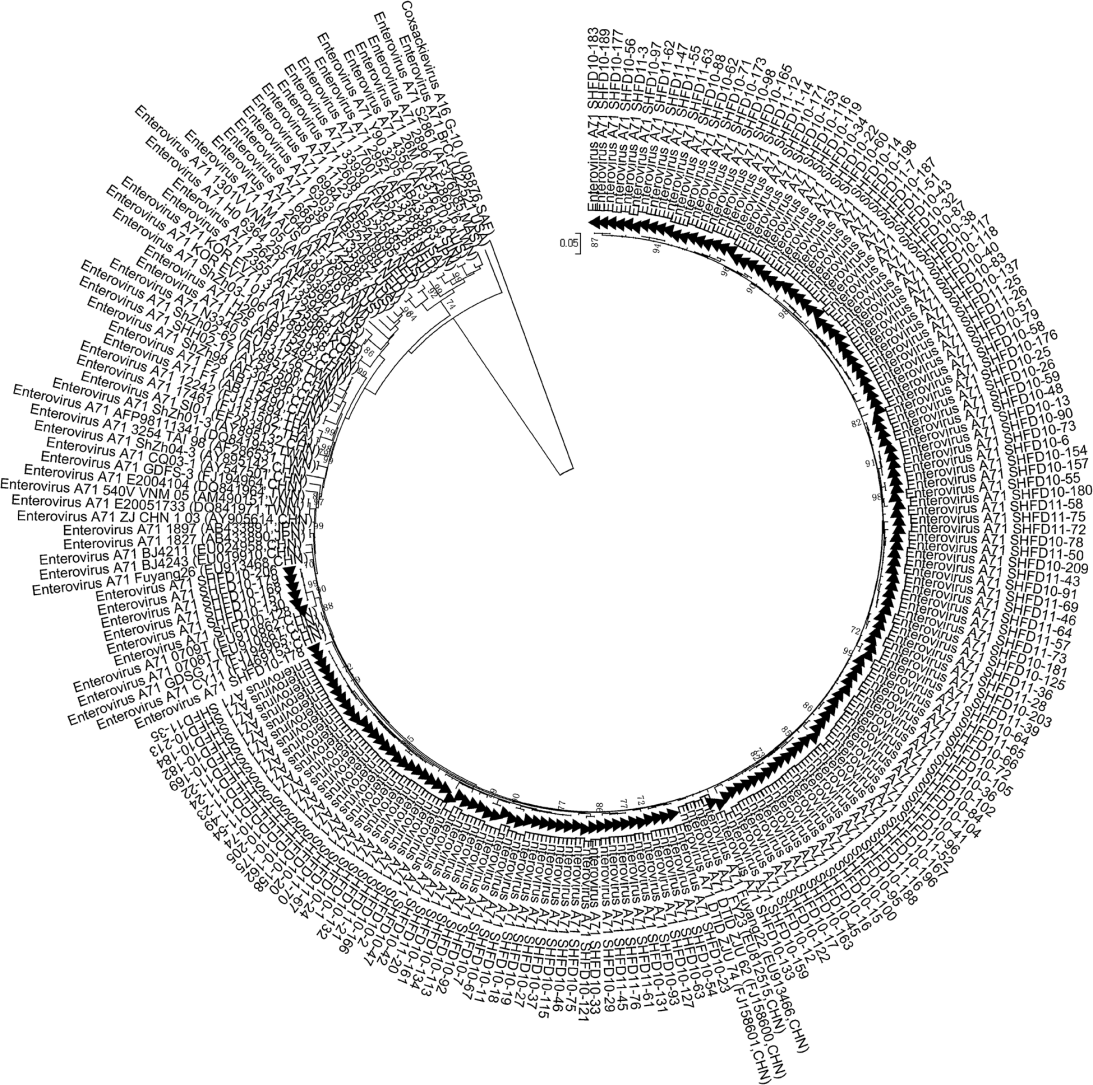
**Phylogenetic analysis with the neighbor-joining method based on the alignment of the 468 nucleotide VP1 gene of 163 Shanghai EV71 strains.** The 59 reference strains were obtained from the US National Center for Biotechnology Information’s Genbank.

The partial VP1 sequence of the 163 EV71 strains from each of the patients was used for phylogenetic analysis. The nucleotide homologies in all the EV71 strains ranged from 90.7% to 100%. The molecular epidemiology of the Shanghai EV71 strains was determined with a phylogenetic dendrogram, with the reference EV71 strains from Genbank representing all the known sub-genotypes (A, B0-B5, C1-C5). The dendrogram showed that all the EV71 strains clustered in the same lineages as a sub-genotype C4. The sequences in sub-genotype C4 could be further divided into C4a and C4b clusters. The Shanghai EV71 strains clustered in C4a, closely related to strains like Fuyang 22, DTID/ZJU-62 which were detected in China in 2008 (Figure 1).

**Figure 2:**
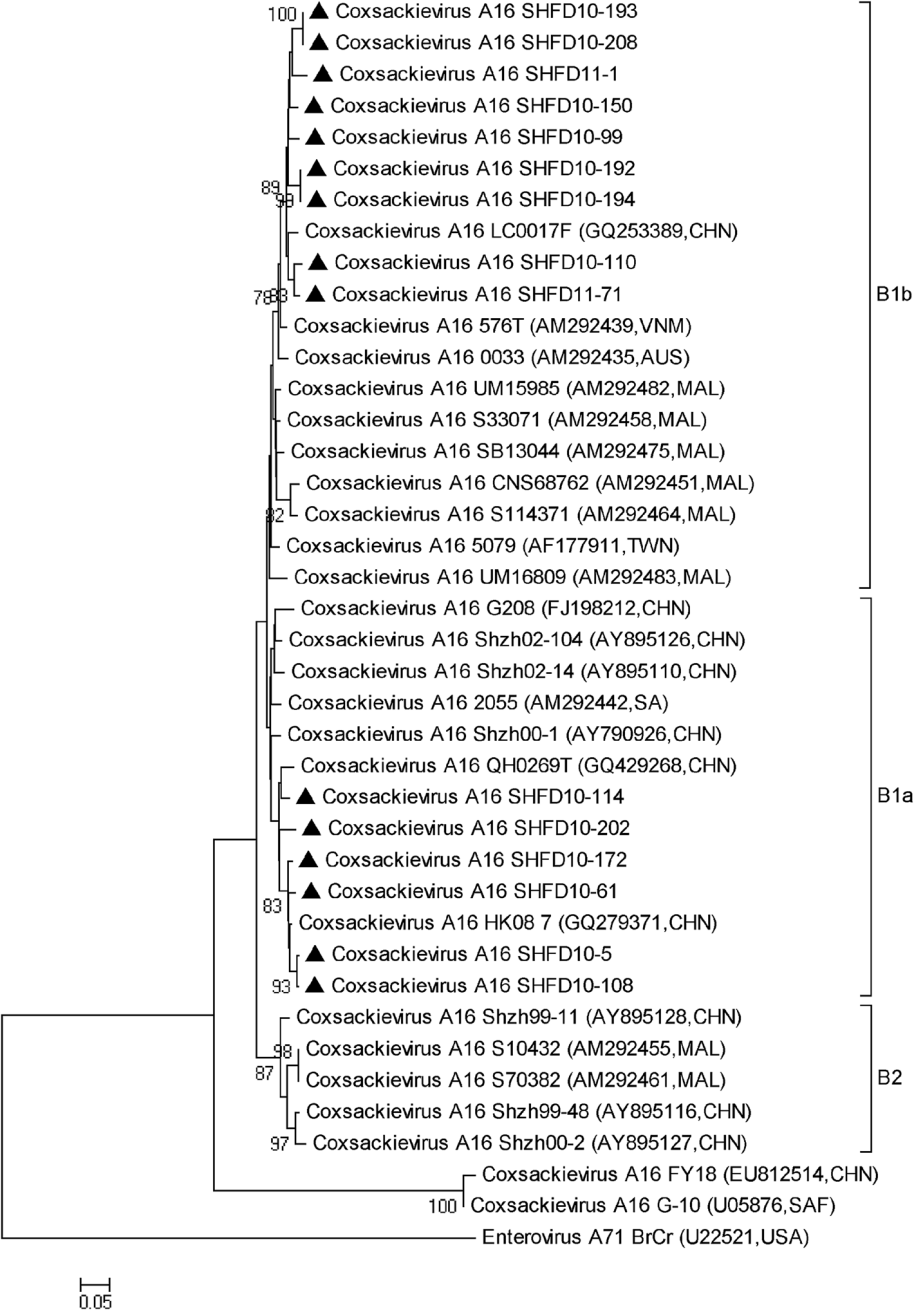
**Phylogenetic analysis based on the alignment of the 488 nucleotide VP1 gene of 15 Shanghai CA16 strains.** The 25 reference strains were obtained from the US National Center for Biotechnology Information’s Genbank.

The nucleotide homologies within all the CA16 strains ranged from 88.4% to 99.1%. Phylogenic analysis of the partial VP1 coding sequences showed all the CA16 strains belonged to the B genotype. Six sequences that were closely related to strains circulating in China (such as HK08-7 and QH0269T) belonged to a B1a cluster, and nine sequences belonged to a B1b cluster, which are closely related to strains identified in Vietnam, Australia, Malaysia and China (Figure 2).

**Figure 3:**
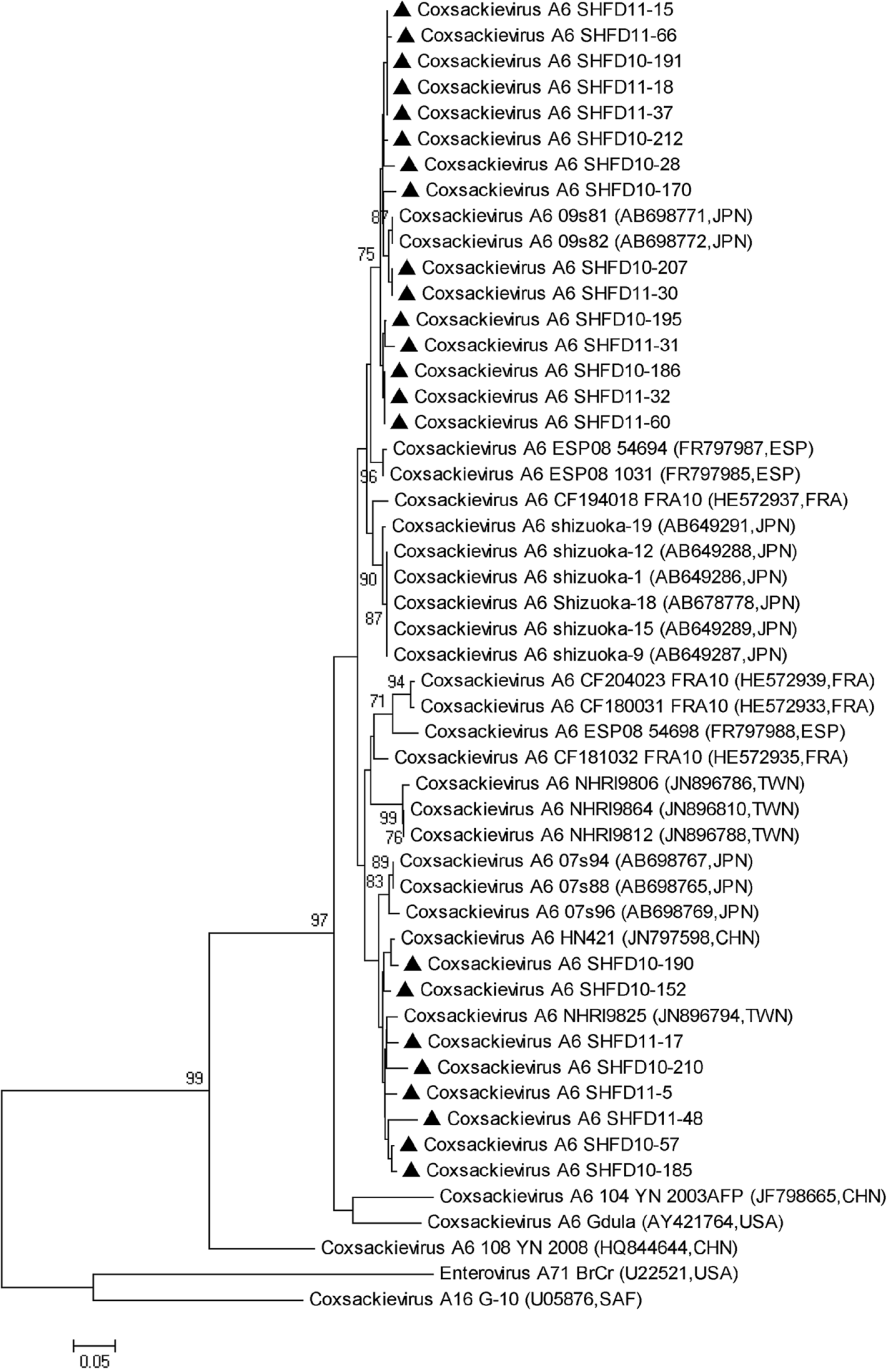
**Phylogenetic analysis based on the alignment of the 248 nucleotide VP1 gene of 23 Shanghai CA6 strains.** The 28 reference strains were obtained from the US National Center for Biotechnology Information’s Genbank.

Of the 23 CA6 partial VP1 gene sequences used for phylogenetic analysis, the nucleotide homologies ranged from 89.8% to 99.0%. The CA6 strains appeared in two branches, 15 of which were closely related to the 09s81 and 09s82 strains detected in Japan in 2009, and another 8 strains were closely related to strains circulating in China and Taiwan (Figure 3).

**Figure 4:**
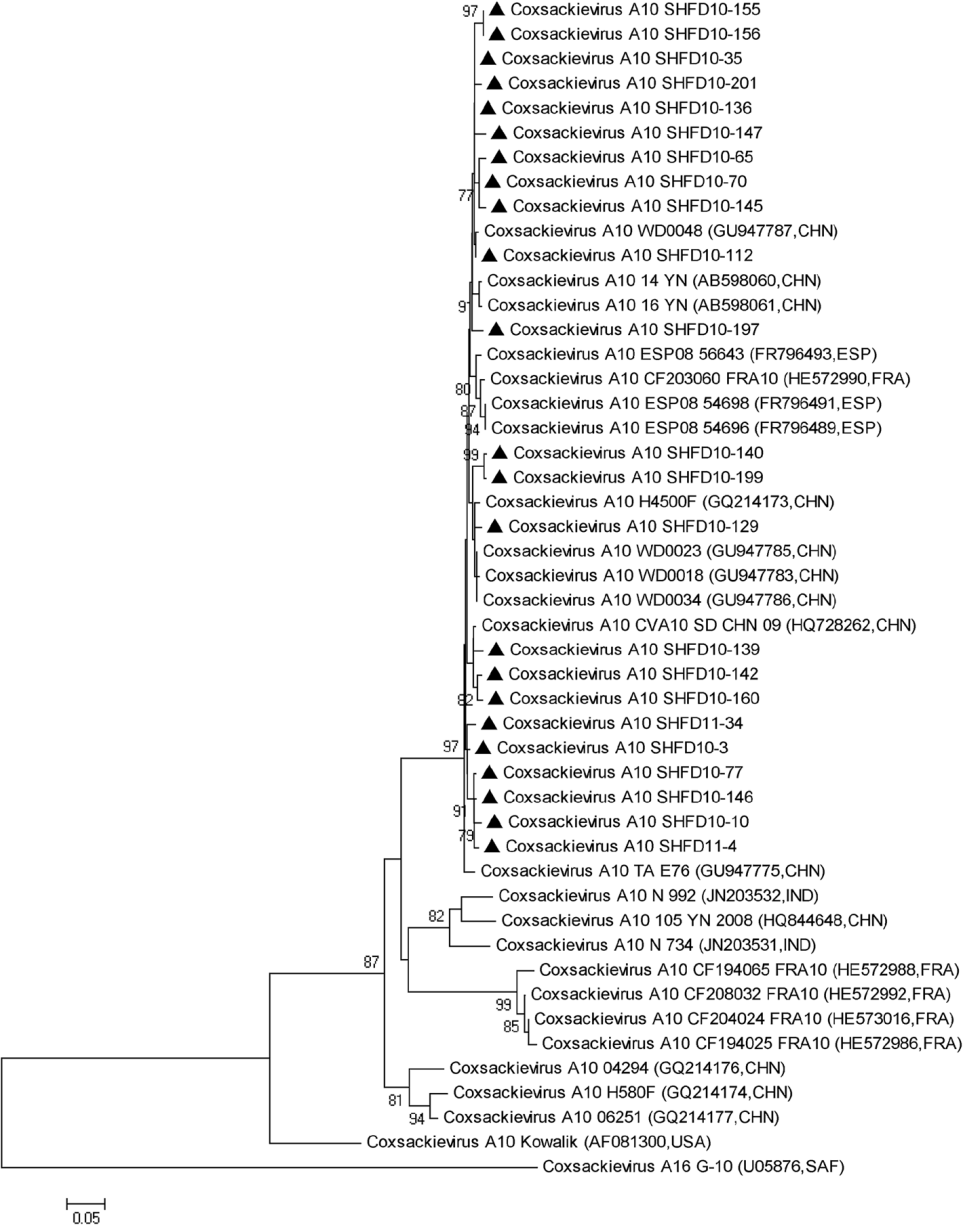
**Phylogenetic analysis based on the alignment of the 355 nucleotide VP1 gene of 23 Shanghai CA10 strains.** The 25 reference strains were obtained from the US National Center for Biotechnology Information’s Genbank.

The nucleotide homologies of CA10 strains ranged from 95.8% to 99.5%. These strains were closely related to the CA10 strains found in China in 2009 and distantly related to the CA10 strain reported in China in 2004 and 2006 and to strains in France and India (Figure 4).

Both the CA12 and CA4 strains were most closely related to the other CA12 and CA4 strains found previously in Jiangsu and Yunnan provinces of China.

### Clinical features of infected patients

All the patients ranged from 6 to 153 months (31.6 ± 19.2M), and 63.1% (183/290) were boys. Almost half the patients had complications such as neurologic, respiratory or circulatory system symptoms.

Compared to patients without EV71 infection, patients with EV71 infection were older (33.8 ± 20.3M vs 27.8 ± 16.3M, *P* < 0.05), had longer hospital stays (4.1 ± 2.4d vs 3.2 ± 1.1d, *P* < 0.05), and higher complication rates (57.8% vs 33.3%, *P* < 0.001).

When we divided patients into 5 groups infected with different enterovirus genotypes, patients with EV71 infection had significantly longer mean hospital stays and a higher incidence of complications than those patients in the CA6, CA10, and CA16 infection groups (*P* < 0.05), but not than those in the CA12 infection group. Patients infected with CA12 were the youngest of the groups and had a higher risk of complications than that of the other non-EV71 infection groups, but the differences were not statistically significant (Table 1).

**Table 1:** Characteristics and symptoms of HFMD patients according to different enterovirus genotypes

**Characteristics**	**EV71**	**CA6**	**CA10**	**CA12**	**CA16**	**Total**
	**(n = 185)**	**(n = 24)**	**(n = 26)**	**(n = 7)**	**(n = 20)**	**(n = 290)**
Age (M)	33.8 ± 20.3	26.4 ± 14.8	25.6 ± 12.2^*^	16.6 ± 7.8^*/#^	33.8 ± 8.5	31.6 ± 19.2
Male, n(%)	119 (64.3)	16 (66.7)	17 (65.4)	6 (85.7)	9 (45)	183 (63.1)
Hospital stay (d)	4.1 ± 2.4	2.9 ± 0.8^*^	2.9 ± 1.0^*^	3.4 ± 1.4	3.3 ± 1.4^*^	3.7 ± 1.5
Fever (>37°C), n(%)	175 (94.6)	20 (83.3)^*^	26 (100)	7 (100)	20 (100)	275 (94.8)
Vomiting, n(%)	72 (39.0)	6 (25)	0 (0)^*^	0 (0)^*^	5 (25)	98 (33.8)
Limb shaking, n(%)	41 (22.2)	1 (4.2)^*^	3 (11.5)	0 (0)	0 (0)^*^	47 (16.2)
Babinski’s sign, n(%) and/or Brudzinski’s sign, n(%)	5 (2.7)	0 (0)	0 (0)	0 (0)	0 (0)	7 (2.4)
Complication, n(%)	107 (57.8)	4 (16.7)^*^	7 (26.9)^*^	3 (42.9)	5 (25)^*^	142 (49.0)

*: Compared with EV71 infection group, *P* < 0.05.

^#^: CA12 infection group compared with CA16 infection group, *P* < 0.05.

## Discussion

In this study, we identified a variety of enterovirus genotypes, including EV71, CA10, CA6, CA16, CA12, CA4, CA14, Echo6, and EV-C, from stool samples of patients hospitalized for HFMD in Shanghai, China. Our results show a diversified pathogen compositions which are similar with reports from other areas of China, Korea and Singapore [13-15].

The most common genotypes in our study all belonged to species A, in which EV71 occupied the predominant position with the highest detection rate of 63.8%. Since the late 1990s, EV71-related HFMD outbreaks have often been reported in the Asia-Pacific region, including Malaysia, Taiwan, Perth, Japan and China [7,19-25]. Thus, EV71 has been identified as the most prevalent genotype causing HFMD in recent years.

EV71 is classified into three genotypes (A, B, and C) on the basis of their phylogenetic relationship of the VP1 structural sequences. At present, the B genotype contains 6 subgenotypes (B0 through B5), whereas the C genotype contains 5 subgenotypes (C1 through C5) [26-32]. The frequency of these subgenotypes differs over time and area. In Malaysia, the prevalent subgenotypes were C1, C2, B3, and B4 in 1997, but C1 and C3 in 2000 and B5 and C1 in 2003 and 2005, respectively [33]. The epidemic genotypes in Taiwan were C2 in 1998 but B4 from 2000 to 2003, C5 in 2006, and B5 in 2008 [34,35].

The predominant subgenotype of EV71 in shanghai during 2010 and 2011 was subgenotype C4, cluster C4a, which is most closely related to the Fuyang22 and DTID/ZJU-62 strains isolated in China. During the past 10 years, the endemic circulation of subgenotype C4 has changed. From 1998 to 2004, EV71 belonged to cluster C4b, but after 2004, cluster C4a replaced C4b and became the predominant virus circulating in China [6,13,36,37]. Our results further confirmed the absolutely dominant position of the EV71 C4a subgenotype in China.

Another major pathogen, CA16, was thought to be associated with mild HFMD without severe neurological complications. The rate of evolution of CA16 strains is far below that of EV71 strains, but sequence information is limited [12]. CA16 has been divided into genogroups A and B, while genogroup B could be further divided into subgenotype B1 and B2 [38]. In our study, B1 was the only prevalent subgenotype, as it was in the CA16 strains in China, Taiwan, Malaysia, Thailand, Vietnam and other areas isolated during 1999 to 2008 [31,39-42].

The higher detection rate of CA6 and CA10, when compared to that of CA16, indicates the importance of these two pathogens in HFMD. Occasional CA6- or CA10-related HFMD outbreaks have been reported. For example, in Singapore, both CA6 and CA10 had a detection rate of 35.3% in a HFMD outbreak in 2008 [14]. The prevalence of CA6 and CA10 was as high as 71% and 28%, respectively, in Finland in 2008, and 28% and 39.9%, respectively, in France in 2010 [43,44]. A CA6-related HFMD outbreak was also reported in Taiwan in 2010 [45]. However, phylogenetic trees analysis was rarely conducted for these two viruses.

In this study, we compared the sequences from our samples to those in Genbank and found that the Shanghai CA6 strain was closely related to strains isolated from Japan, Taiwan, and China. The Shanghai CA6 strain belonged to two evolutionary clusters with a high nucleotide homology, whereas all the CA10 strains were most closely related to the strains found in the Shandong and Yunnan provinces of China.

Although not prevalent, CA4 and CA12 were identified as pathogens attributing to HFMD in Shanghai. CA4 caused a high infection rate in preschool children in Taiwan from 2006 to 2008, whereas only one case of CA4 infection were reported in Korea in 2009 [14,41]. Sporadic HFMD cases with CA12 infection were seen in other areas of China between 2008 and 2009 [13,46,47].

Most enterovirus infections are self-limited and do not require hospitalization, but EV71 infection in young children frequently cause complications and can progress quickly [3,48,49]. However the clinical characteristics of enterovirus genotypes, other than EV71 and CA16, have not been well studied. We found that HFMD cases infected with CA6, CA10 and CA16 caused less complications compared to those infected with EV71. But worth of note, the patients infected with CA12 had the youngest age of onset and most likely have the highest incidence of complications than any of the other non-EV71 infection groups. Since there were only 7 cases in this group, more study and data are needed for accurately identifying the pathogenetic characteristic of CA12 infections in China. In addition, we did not observe the clinical symptoms, such as skin ulceration and an obvious onychomadesis, that were associated with CA6-caused HFMD in Finland, Taiwan and Spain [45,50,51].

One limitation of this study is that different samples were not taken in the patients i.e. throat swab, vesicle and CSF which may influence the integrality of the data and bring bias to the conclusion. Due to the retrospective nature of the study, stools were the only specimen sent routinely to virology laboratory for enterovirus testing and collection of more types of samples could not be performed. However, it is widely recognized that HFMD is a common disease of children mostly associated with the human enterovirus species A. All the stools were from hospitalized patients clinically diagnosed with HFMD, and all the specimens were collected during the acute phase of the illness. Thus we think that the enterovirus identified in the stool specimen could represent the pathogen causing HFMD and had a great relationship with the clinical symptoms. In the future, to elucidate further the epidemiology of the pathogens for HFMD, a prospective study will be developed and multiple clinical specimens from the same patient need to be taken and evaluated.

## Conclusions

In summary, this study provides useful epidemiological data on the features of the pathogen compositions of HFMD as well as clinical characteristics differing in enterovirus genotypes in Shanghai, China. It deserves our attention that early identification of enterovirus genotypes is important for diagnosis and treatment of HFMD patients.

## Abbreviations

HFMD: Hand foot and mouth disease; EV: Enterovirus; EV71: Enterovirus 71; CA16: Coxsackievirus A16; NCBI: US national center for biotechnology information; DEPC: Diethypyrocarbonate.

## Competing interests

The authors declare that they have no competing interests.

## Authors’ contributions

JX designed the study and contributed to manuscript writing. MX perfromed the experiment, analyzed the data and wrote the manuscript. LS, LC, ND and HZ contributed to specimen collection, reagents preparation, setting up the assay and data analysis. All authors have read and approved the final manuscript.

## Pre-publication history

The pre-publication history for this paper can be accessed here:

http://www.biomedcentral.com/1471-2334/13/489/prepub
